# Supplementary figures and images for: LPS induces inflammatory chemokines via TLR-4 signalling and enhances the Warburg Effect in THP-1 cells
Source: PLoS One. 2019 Sep 27;14(9):e0222614. doi: 10.1371/journal.pone.0222614 (PMC6764657; doi:10.1371/journal.pone.0222614)

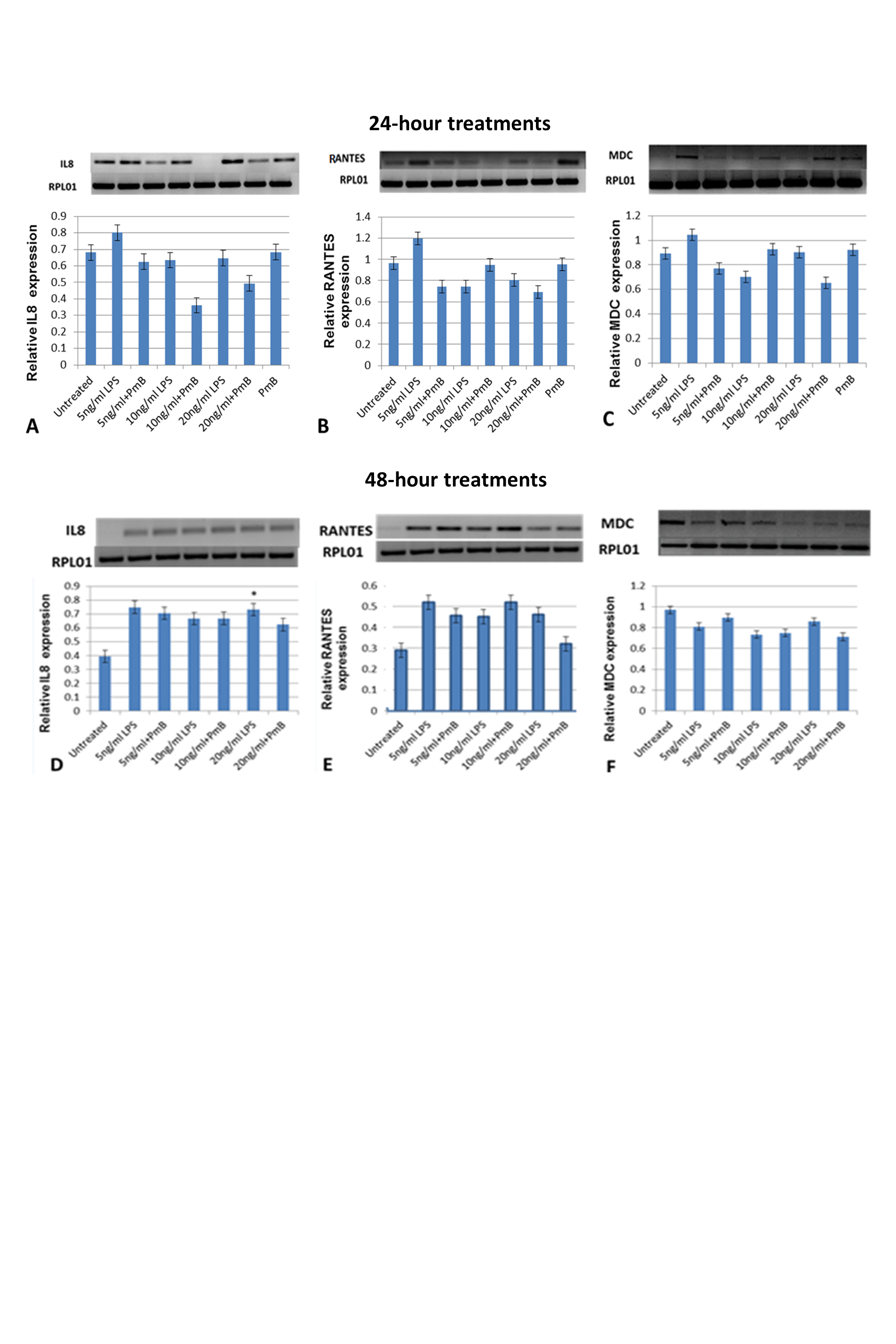

Supplement: S1 Fig — A and D show RT-PCR gel images and corresponding densitometry results for IL8, B and E: RANTES and C and F: MDC, following 24-, and 48-hour treatments respectively, with 5, 10 and 20 ng/ml of LPS and co-treatments with 10 μg/ml PmB. All genes were normalised with reference, RPL01. (TIF) [file pone.0222614.s001.tif]

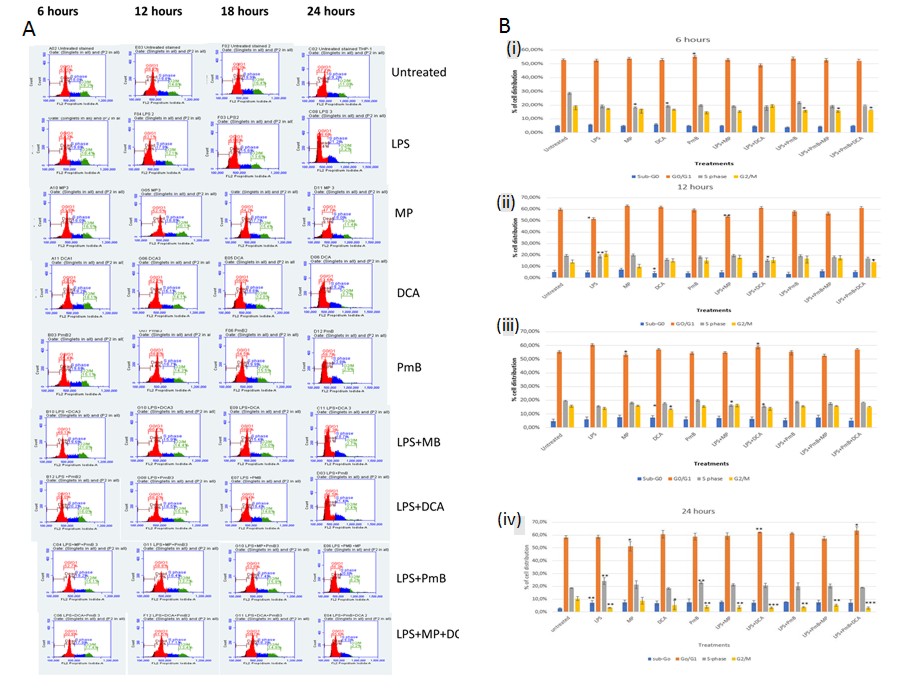

Supplement: S2 Fig — The cells were treated with 5 ng/ml LPS (Lipopolysaccharides), 10 mM DCA (dichloroacetate), 0.08% MP (Methyl pyruvate) and/or 10 μg/ml PmB (Polymyxin B) for 6, 12,18, and 24 hours. Sub G0/G1 phase is shown in maroon; G0/G1 phase in red, S-phase in blue, and G2/M phase in green. The cell cycle assay was performed using BD Acurri TM flow cytometer. The data represented here is a representative of three separate experiments. Florescence data were acquired on the FL2 (orange fluorescence) channel. B. Cell cycle analysis in THP-1 cells. Cells were treated with 5 ng/ml of LPS, 0.08 MP (methyl pyruvate), 10 mM DCA (dichloroacetate) and/or 10 μg/ml PmB for 6, 12, 18 and 24 hours (i)–(iv). The blue bars represent sub-G0/G1 cell populations; orange bars show G0/G1 cells, grey bars indicate S-phase populations and yellow bars depict G2/M cell populations. The data are represented as mean ± SD from 3 independent experiments (* indicates p <0.05, ** indicates p <0.01, *** indicates p <0.001. All statistics were computed using GraphPad Quick Calcs software. (JPG) [file pone.0222614.s002.jpg]

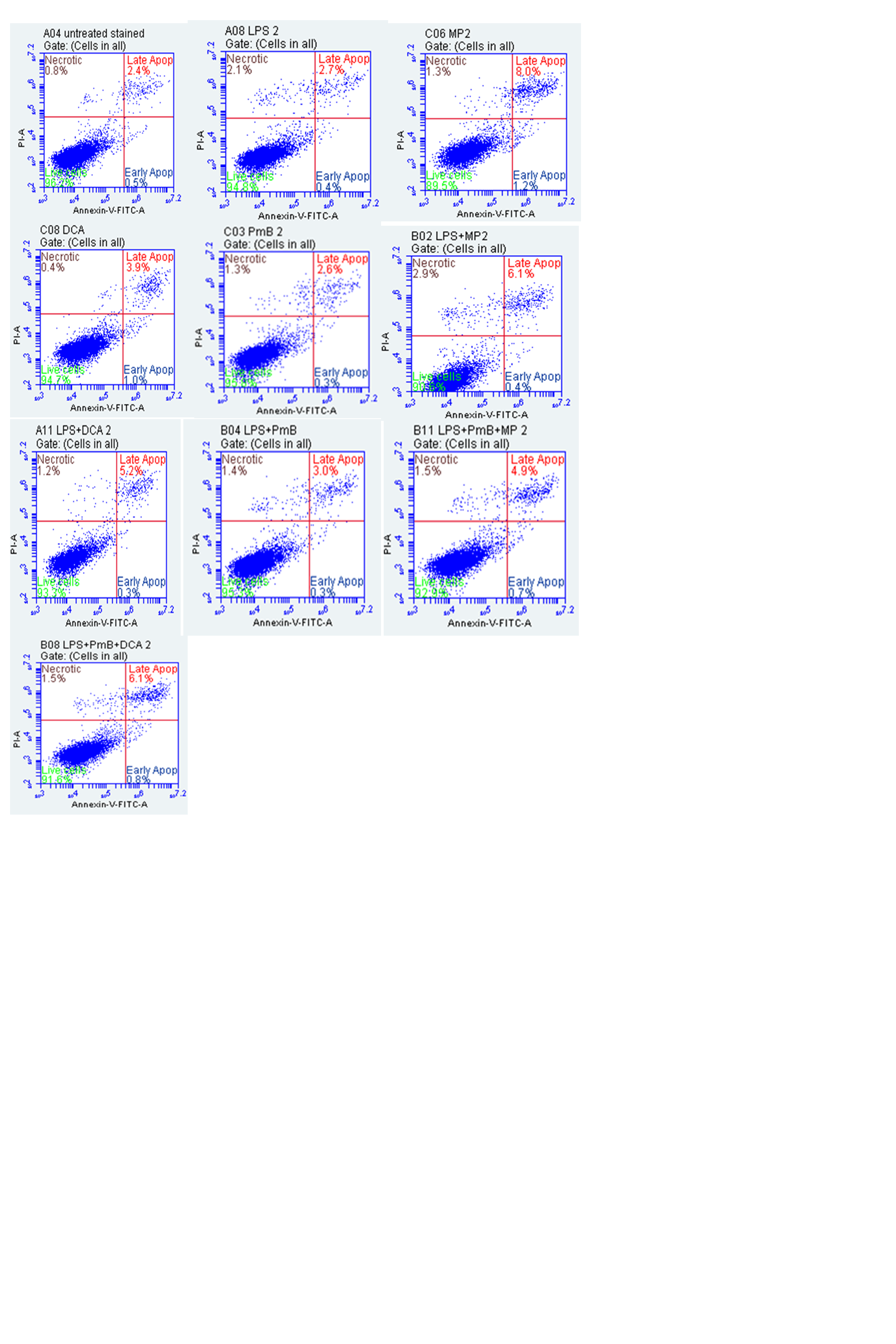

Supplement: S3 Fig — Each diagram represents a treatment. Annexin V/PI stained THP-1 cells following treatment with either 5 ng/ml LPS, 0.08% MP, 10mM DCA,10 μg/ml PmB and combination of these treatments in comparison with untreated cells for 24 hours. Each quadrant represents populations of viable (lower left), early apoptotic (lower right), late apoptotic (upper right) and necrotic (upper left) cells The data were acquired using a BD Acuri C6 flow cytometer with propidium iodide (PI) fluorescence monitored on the FL3 (red fluorescence) channel (shown on the y-axis) while annexin V-alexa 488 of the FL1 (green fluorescence) channel (shown on the x-axis). (TIF) [file pone.0222614.s003.tif]

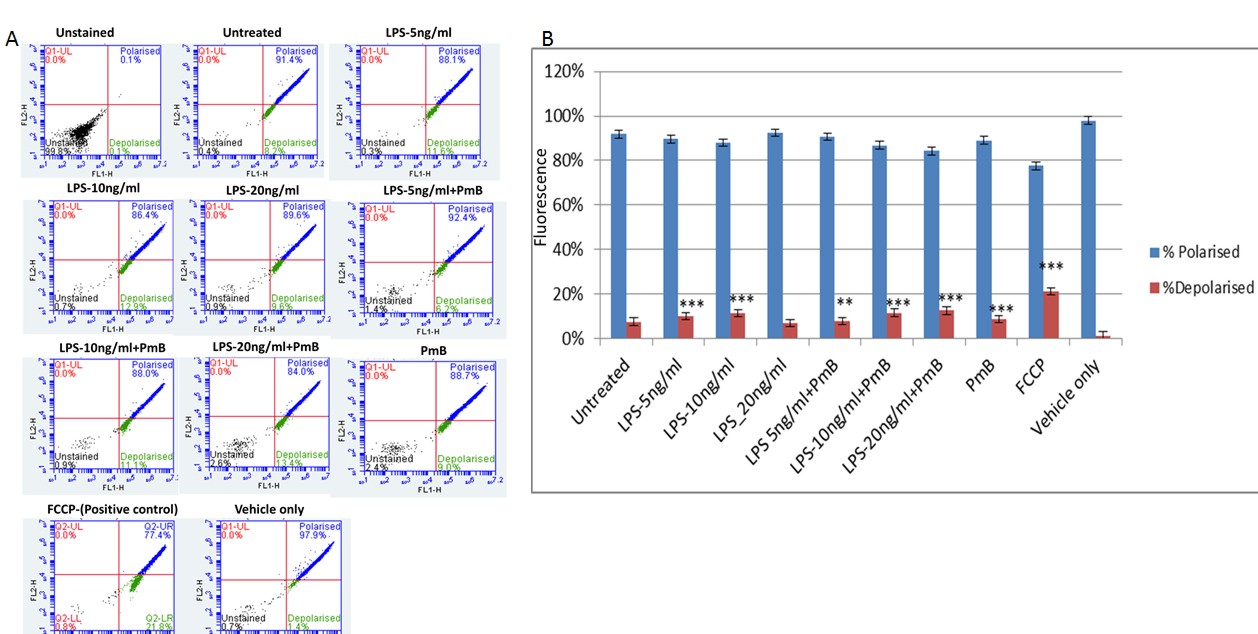

Supplement: S4 Fig — A Each diagram is a representative of three independent treatments. The cells were treated with 5, 10 and 20 ng/ml LPS and/or polymyxin B for 48 hours. The x-axis represents the FL2 (Green fluorescence) channel, while the y-axis shows FL2 (orange fluorescence) channel. The lower left quadrant shows unstained cells, lower right quadrant: green fluorescent (depolarised) cells; and the upper right quadrant: orange fluorescent (polarised) cells. FCCP (Carbonyl cyanide-4-(trifluoromethoxy) phenylhydrazone), dissolved in 95% ethanol was used as positive control. 95% ethanol was used as the vehicle control. B. MMP analysis showing significant membrane depolarisation following LPS treatment. The histogram shows mitochondrial membrane depolarisation following treatments with LPS (5, 10 and 20 ng/ml) and co-treatments with polymyxin B (PmB). Fluorescence intensity to assess mitochondrial membrane potential was monitored using FL1 (green) and FL2 (orange) fluorescent channels in a BD Acuri C6 flow cytometer. The blue bars show polarised cells while the red bars show the percentage of depolarised cells. A Student t-test was used to generate the p-values which compared the difference between the untreated and treated sample values. The data is represented as mean ± SD from 3 independent experiments (* indicates p <0.05, ** indicates p <0.01, *** indicates p <0.001). GraphPad Quick Calcs software was used to compute all statistics. (JPG) [file pone.0222614.s004.jpg]
