# Supplementary material for: LPS induces inflammatory chemokines via TLR-4 signalling and enhances the Warburg Effect in THP-1 cells
Source: PLoS One. 2019 Sep 27;14(9):e0222614. doi: 10.1371/journal.pone.0222614 (PMC6764657; doi:10.1371/journal.pone.0222614)
Supplement: Attachment — Submitted filename: Response to Reviewers.doc.docx [file pone.0222614.s005.docx]

**Response to reviewers**

Review Comments to the Author

*Please use the space provided to explain your answers to the questions above. You may also include additional comments for the author, including concerns about dual publication, research ethics, or publication ethics. (Please upload your review as an attachment if it exceeds 20,000 characters)*

**Reviewer #1:**

1. **I assume fig. 1a is the western blot for TLR protein. It does not seem upon induction with LPS 5ng/ml the protein does not increased while mRNA increased. How many repeats were carried out for western blot and if possible quantify after three repeats, if done.**

**Fig 1a** is an RT-PCR showing TLR4 mRNA expression and not a Western blot. The reviewer must have made a mistake because the legend clearly indicates this. We have highlighted the section in Figure 1 legend that shows that the blots depict RT-PCR. No repeats were performed for TLR4 expression since it is well-known that THP-1 cells express TLR4. This was just to confirm expression and induction by LPS at the low concentrations compared to published ones. The error bars were generated accidentally because the “error bars” item was ticked off on the spreadsheet “Chart Elements” menu. We apologise for this. We generated a new graph and presented it in **Fig 1a**.

2**. No mention of Western blot protocol in material method section. Authors has to be careful in this regard and update it.**

We did not perform any Western blot experiments. Hence, no Western blot protocol was included.

**3. Fig. 3a- I am not sure how many repeats were carried out for ELISA**

The ELISA assay was performed in a single experiment as a screening tool as stated in the results and discussion section of the manuscript. We validated this expression profile by using RT-PCR in two biological replicates. We are convinced about the validity of the Array result because the RT-PCR expression profile is consistent with the Array.

**4. Fig. 3b-e – Again for western blots- I would like to see how many repeats were carried out and if possible can they quantify the western blots that can be comparable with the RT-PCR result.**

No Western analyses were conducted in this submission. The legend of **Figure 3** indicates that there were two biological replicates (highlighted).

**5. This is because it is confusing for all the Figures in 3, as an example – the difference between mRNA to protein in Fig. 3b- compared to 5ng/ml+PmB to 10ng/ml LPS the mRNA level is same between these samples while the 10ng/ml LPS the protein level is high.**

No Western analyses were conducted in this submission. The source of the confusion could be the lack of explanation for the bar graphs. We have now included in the legend that the lower panels represent quantification of the RT-PCR gels bands. They are not protein measurements.

**6. Similarly, for RANTES (Fig. 3C)- as by visualization 10ng/ml LPS has protein while 10ng/ml LPS with PmB no protein while the mRNA profile is different between these samples.**

No Western analyses were conducted in this submission. Please see (5) above.

**7. Thus, it is important to address this issue upon repeating western for all these genes (Fig. 3c to e) and quantify whether there is any mRNA and protein difference.**

No Western analyses were conducted in this submission. Please see (5) above.

**8. Since the authors have addressed the impact of cytokines and its linkage with downstream pathways. Why not address few genes that are linked to IL8 or RANTES by knocking down these genes to show its effect on its interactome?**

We do agree that this study reveals a broad view and complexity of the molecular pathways induced by infection in the tumour microenvironment. However, at this stage the primary questions were about nature of the Warburg Effect in monocytes. The finer studies suggested by the reviewer are being considered for new studies where the tumour microenvironment is simulated in 3D cultures. We do appreciate these comments because they reinforce our views about the future experiments.

**9. I would also like to know NF kappa B expression upon these treats because it will be an important marker to show that even though NF kappa B expression increased in LPS treatment, only few of its target cytokines expression is altered.**

NF Kappa B regulates multiple aspects of innate immunity and has two broad mechanisms: the (i) canonical and (ii) non-canonical pathways. It is not too surprising that not all effectors known to be mediated by it are induced as its activity is contextual (temporal and cell specific). It is, however, notable that in the profile we present, the chemokines (known to be NF-Kappa B targets) are induced but the cytokines (some known known to be NF Kappa B targets) are poorly induced or not at all). We have addressed the NF kappa B influence in the discussion section. Although we appreciate the interest of the reviewer in NF kappa B expression, we do not think that a focussed study on NF kappa be would be useful in understanding the Warburg Effect at this stage. It could probably enlighten us about cytokine expression pathways – which is not the primary interest of the manuscript.

**Reviewer #2**:

**In this manuscript, the authors analyzed cytokine production and Warburg effect induced by LPS in THP-1 AML cell line. While it is already widely known that LPS induces inflammatory cytokine production, the authors report an interesting finding in the glycolytic changes in THP-1 cells. The techniques used by the authors are sound, and the results fully demonstrate their conclusion.**

We are grateful for these comments.
